# Supplementary material for: Molecular Epidemiology, Virulence Traits and Antimicrobial Resistance Signatures of Aeromonas spp. in the Critically Endangered Iberochondrostoma lusitanicum Follow Geographical and Seasonal Patterns
Source: Antibiotics (Basel). 2021 Jun 22;10(7):759. doi: 10.3390/antibiotics10070759 (PMC8300795; doi:10.3390/antibiotics10070759)
Supplement: Supplementary file 1 [file antibiotics-10-00759-s001.zip › Supplementary material 1.pptx]

## Slide 1
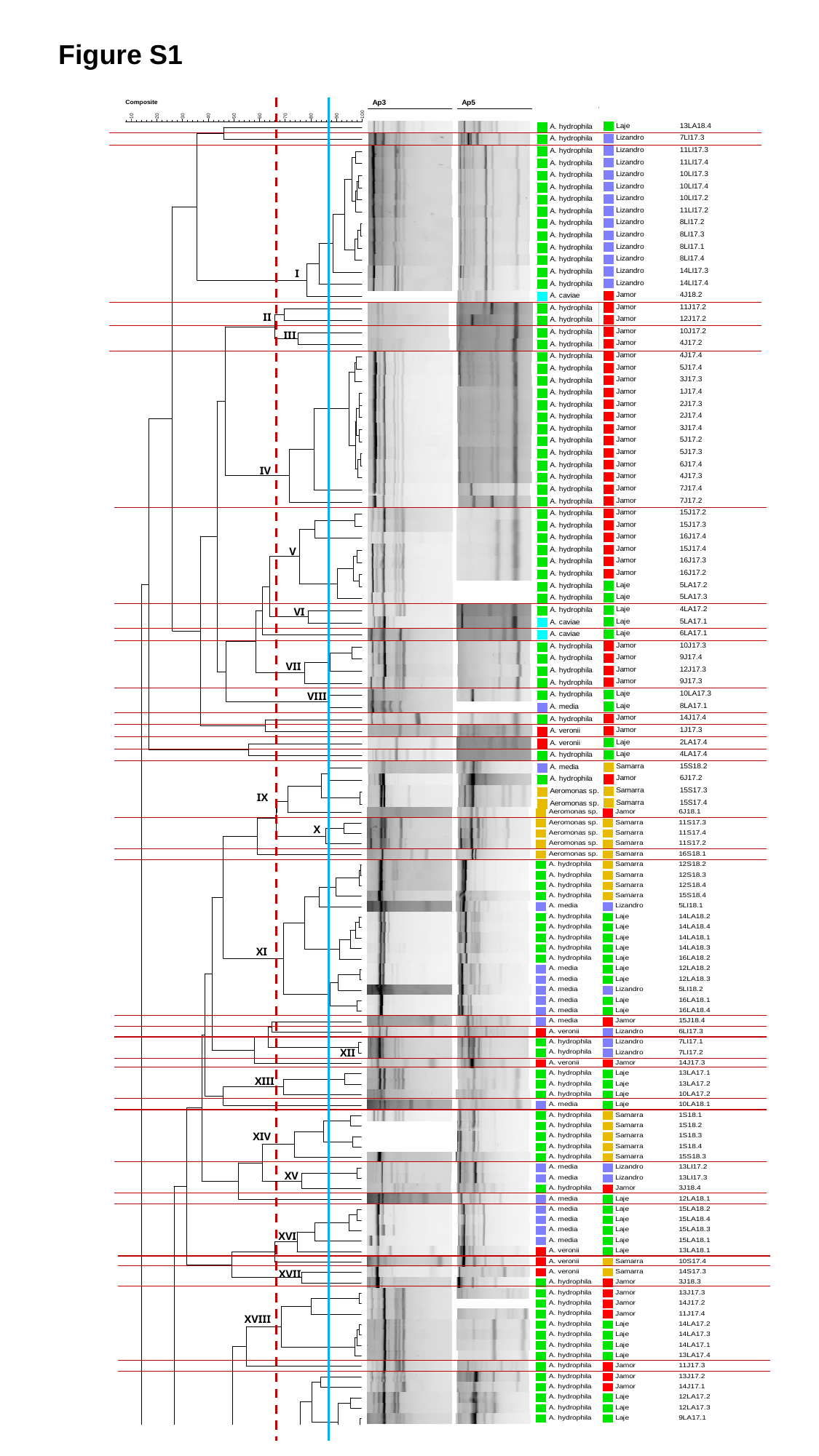

Figure S1
I
II
III
IV
V
VI
VII
VIII
IX
X
XI
XII
XIII
XIV
XV
XVI
XVII
XVIII

## Slide 2
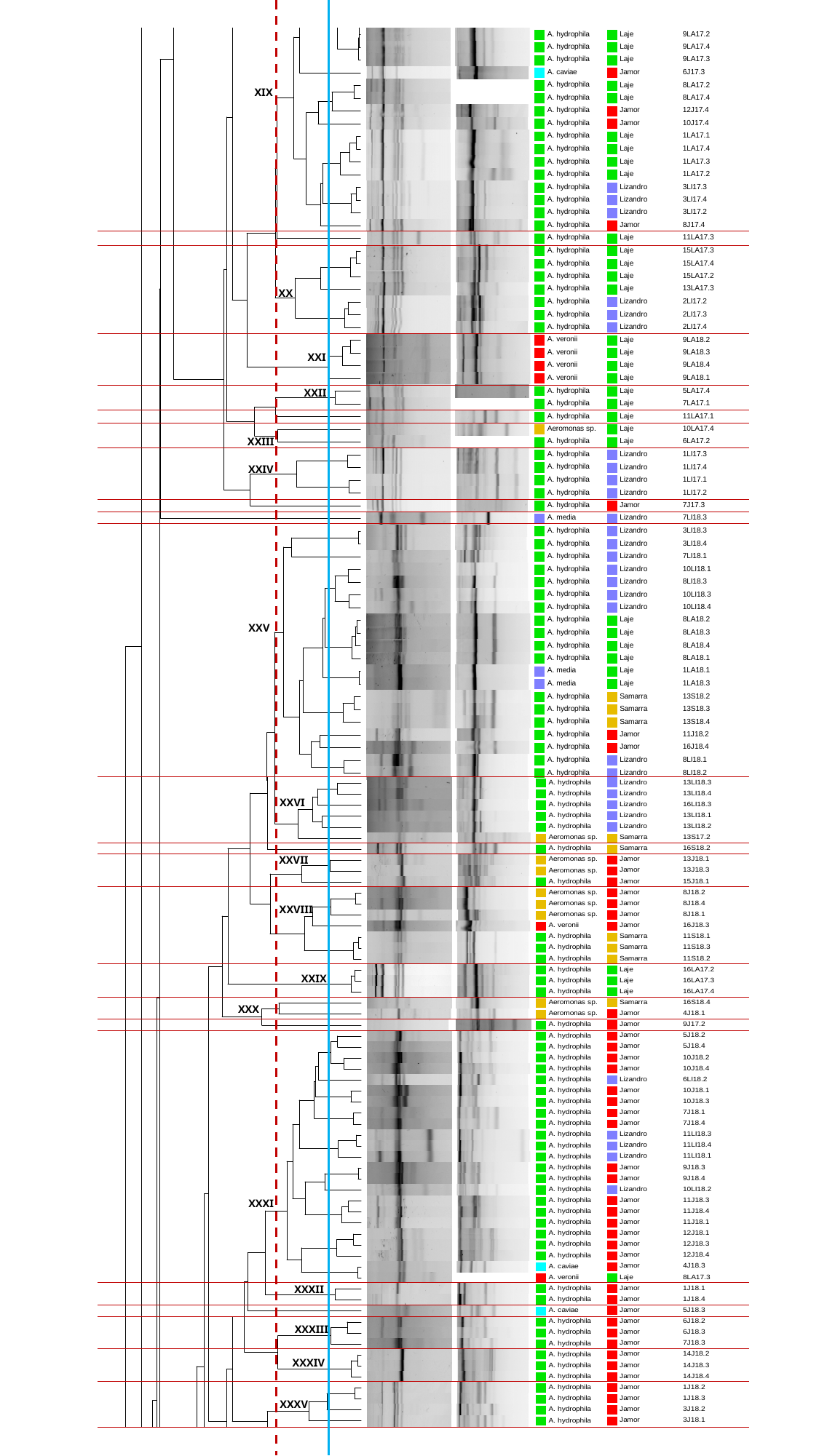

XIX
XX
XXI
XXII
XXIII
XXIV
XXV
XXVI
XXVII
XXVIII
XXIX
XXX
XXXI
XXXII
XXXIII
XXXIV
XXXV

## Slide 3
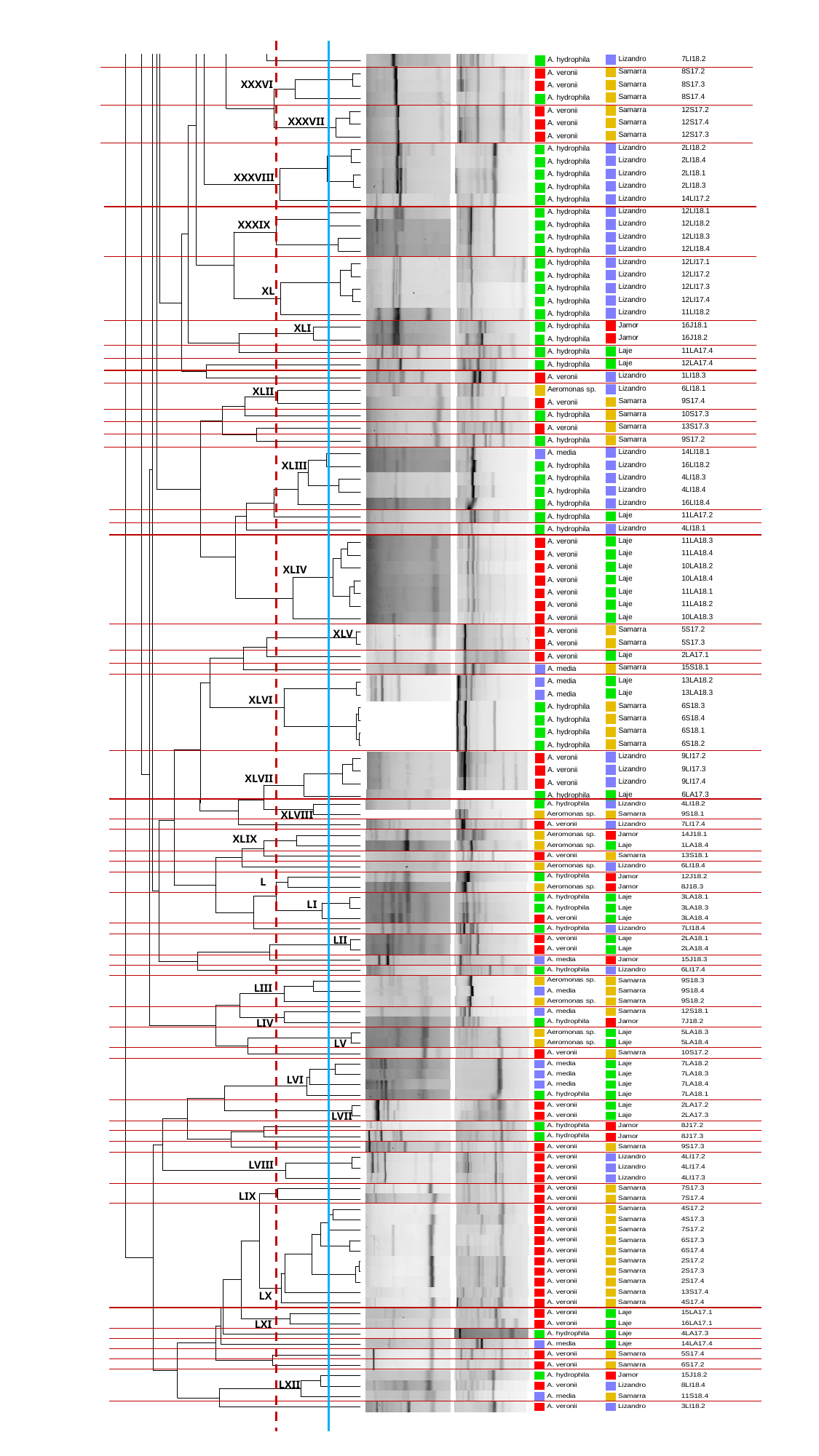

XXXVI
XXXVII
XXXVIII
XXXIX
XL
XLI
XLII
XLIII
XLIV
XLV
XLVI
XLVII
XLVIII
XLIX
L
LI
LII
LIII
LIV
LV
LVI
LVII
LVIII
LIX
LX
LXI
LXII
