# Supplementary material for: Molecular Epidemiology, Virulence Traits and Antimicrobial Resistance Signatures of Aeromonas spp. in the Critically Endangered Iberochondrostoma lusitanicum Follow Geographical and Seasonal Patterns
Source: Antibiotics (Basel). 2021 Jun 22;10(7):759. doi: 10.3390/antibiotics10070759 (PMC8300795; doi:10.3390/antibiotics10070759)
Supplement: Supplementary file 1 [file antibiotics-10-00759-s001.zip › Supplementary material 2.pptx]

## Slide 1
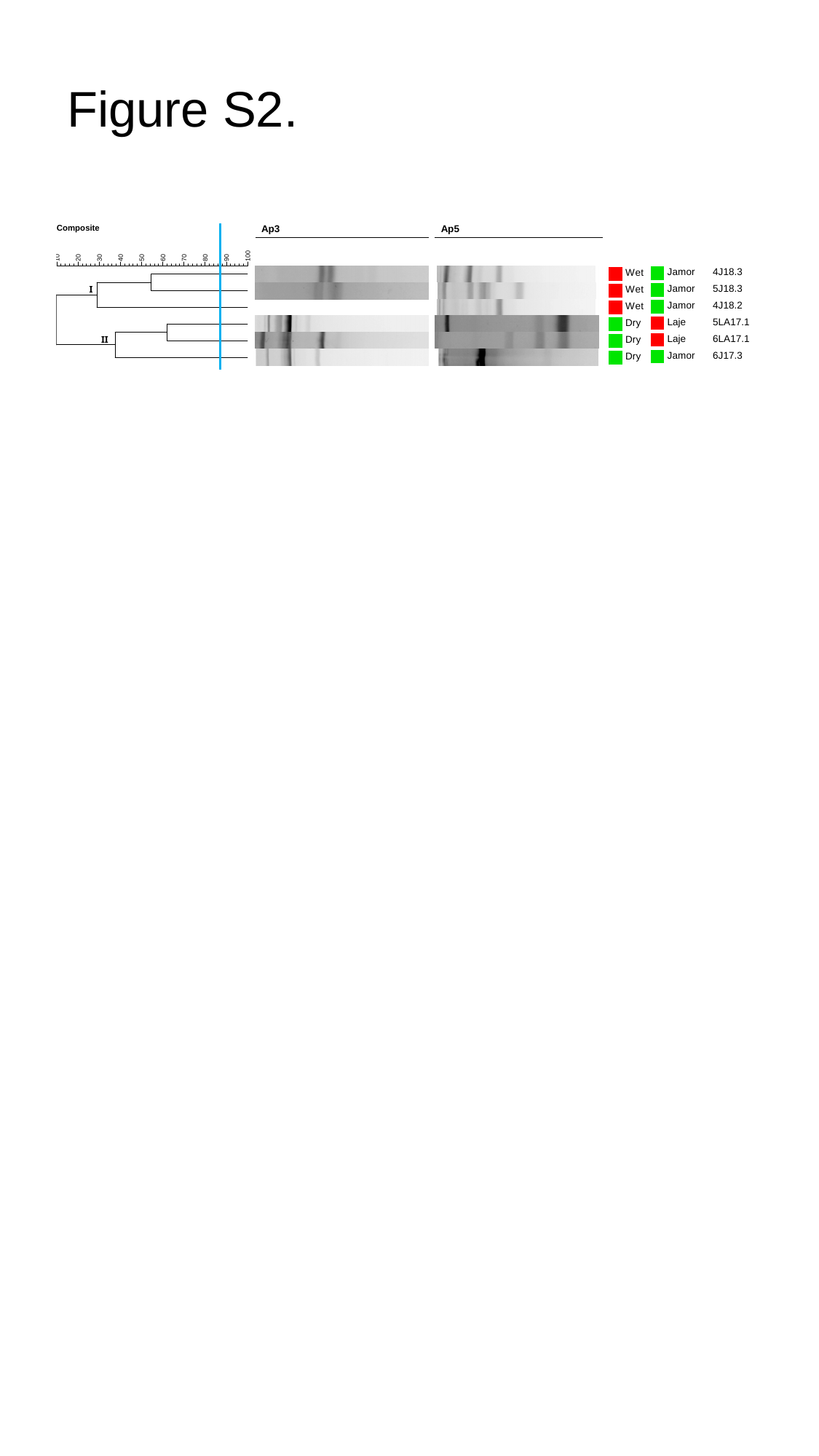

# Figure S2.
I
II

## Slide 2
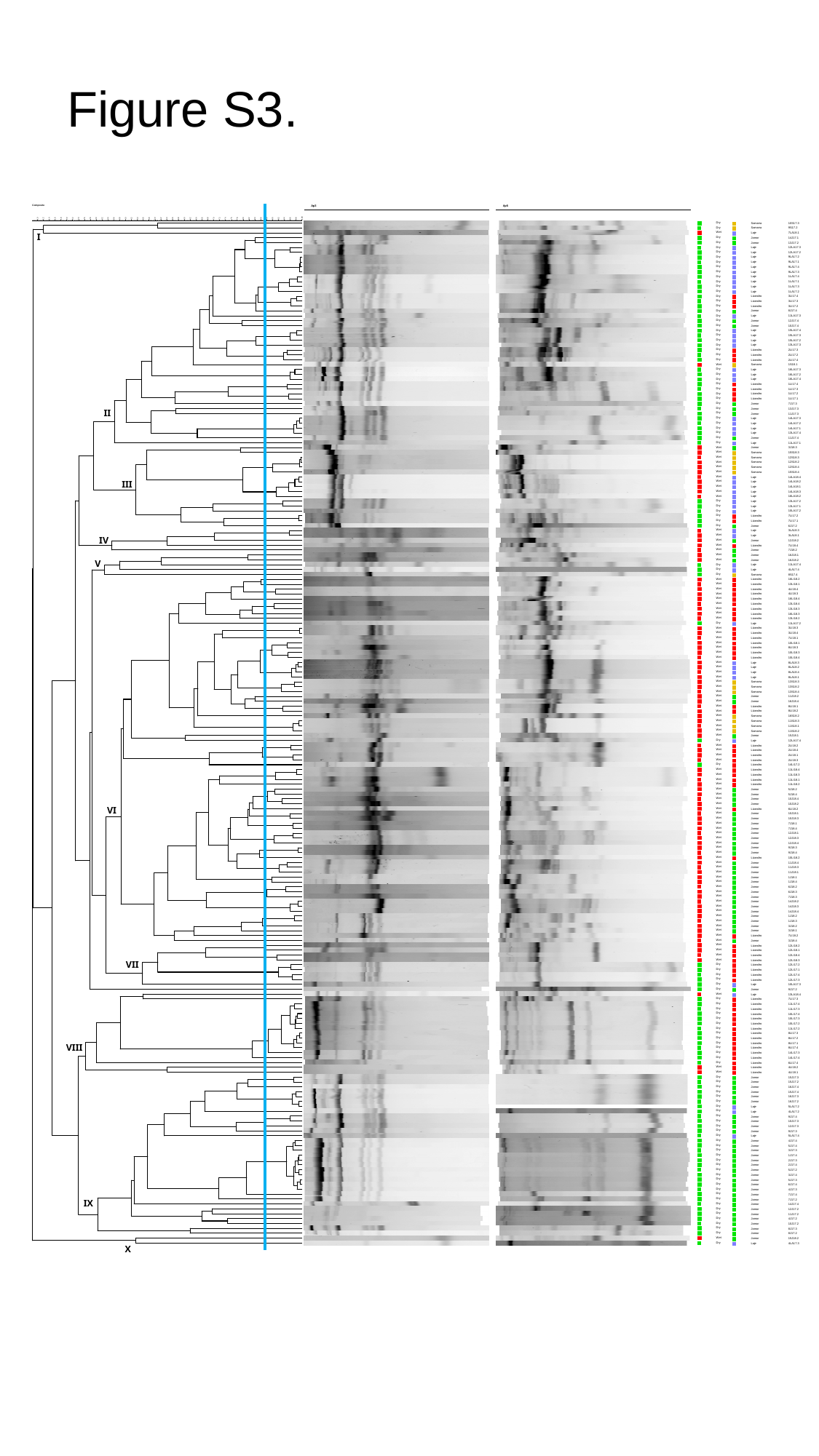

# Figure S3.
I
II
III
IV
V
VI
VII
VIII
IX
X

## Slide 3
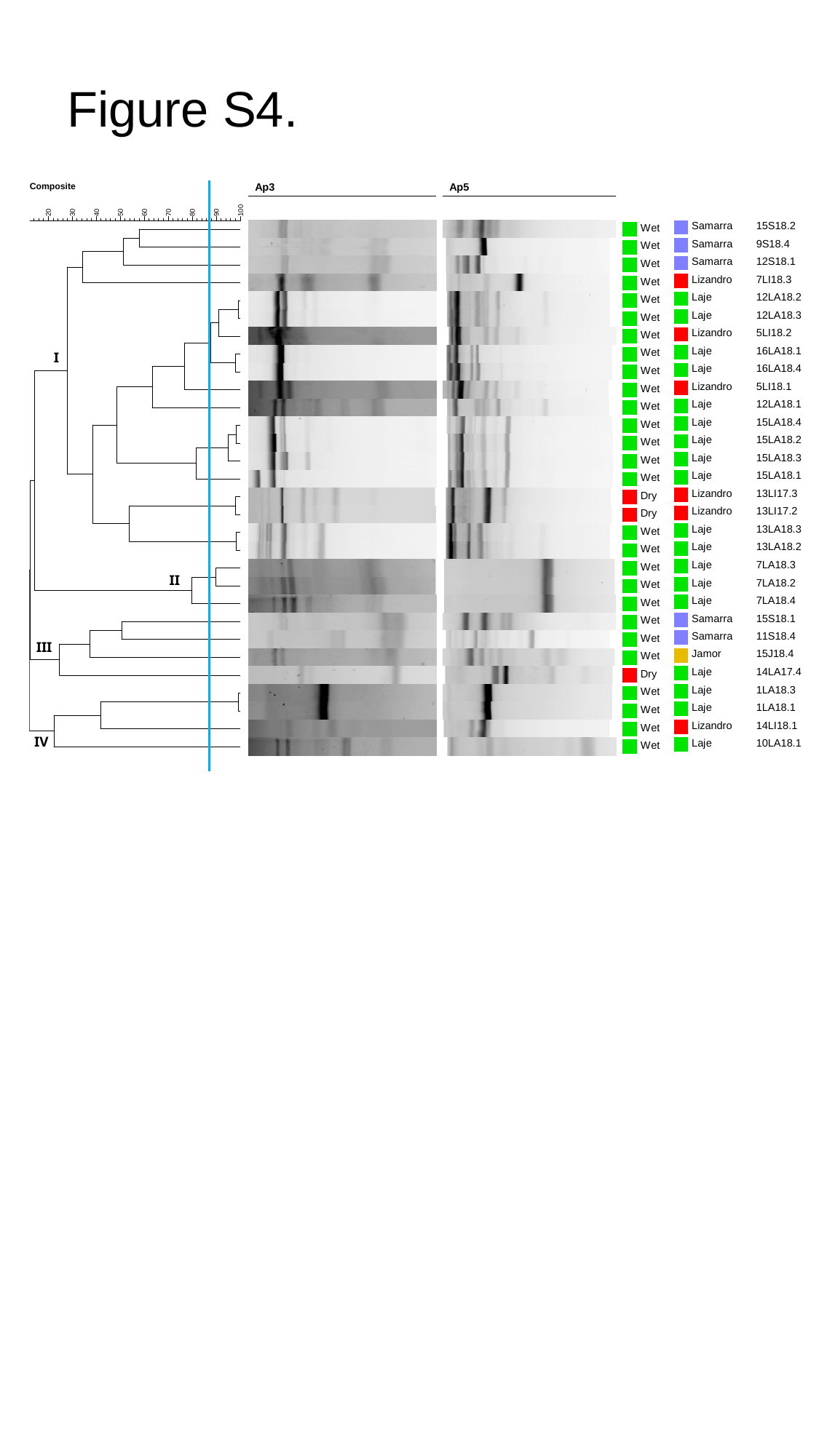

# Figure S4.
I
II
III
IV

## Slide 4
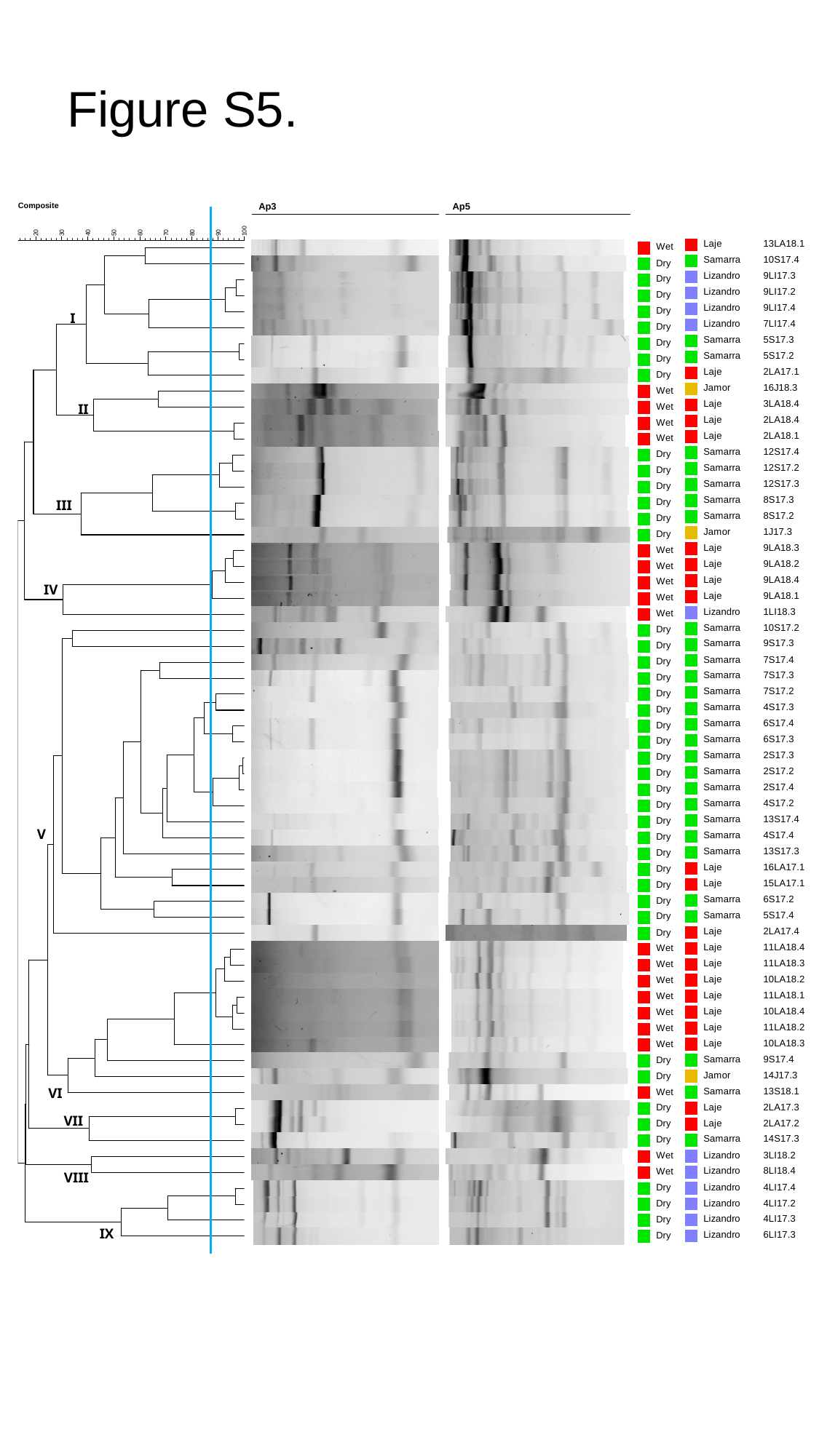

# Figure S5.
I
II
III
IV
V
VI
VII
VIII
IX
