# Supplementary material for: Molecular Epidemiology, Virulence Traits and Antimicrobial Resistance Signatures of Aeromonas spp. in the Critically Endangered Iberochondrostoma lusitanicum Follow Geographical and Seasonal Patterns
Source: Antibiotics (Basel). 2021 Jun 22;10(7):759. doi: 10.3390/antibiotics10070759 (PMC8300795; doi:10.3390/antibiotics10070759)
Supplement: Supplementary file 1 [file antibiotics-10-00759-s001.zip › Supplementary material 3.pptx]

## Slide 1
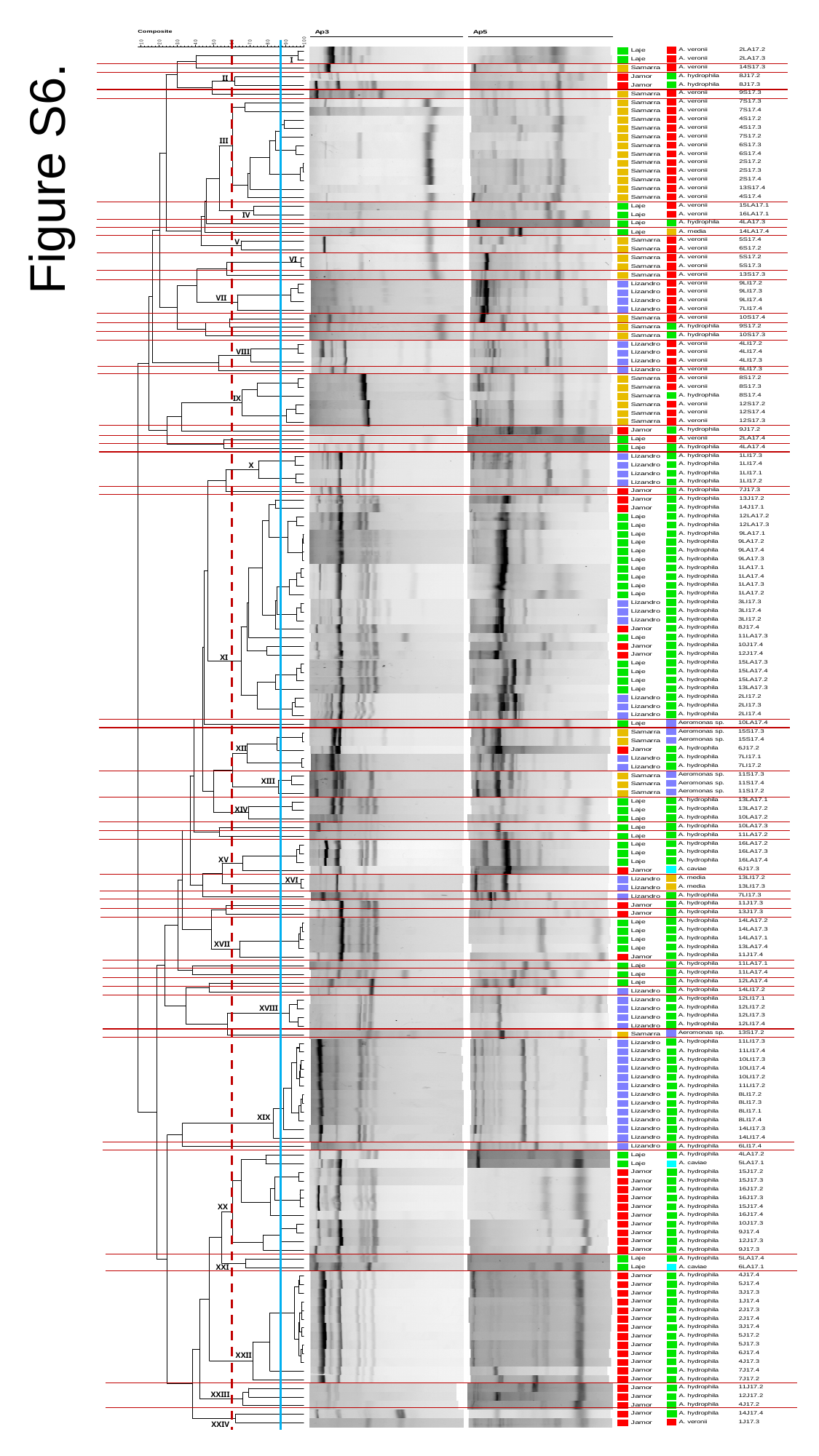

I
II
# Figure S6.
III
IV
V
VI
VII
VIII
IX
X
XI
XII
XIII
XIV
XV
XVI
XVII
XVIII
XIX
XX
XXI
XXII
XXIII
XXIV

## Slide 2
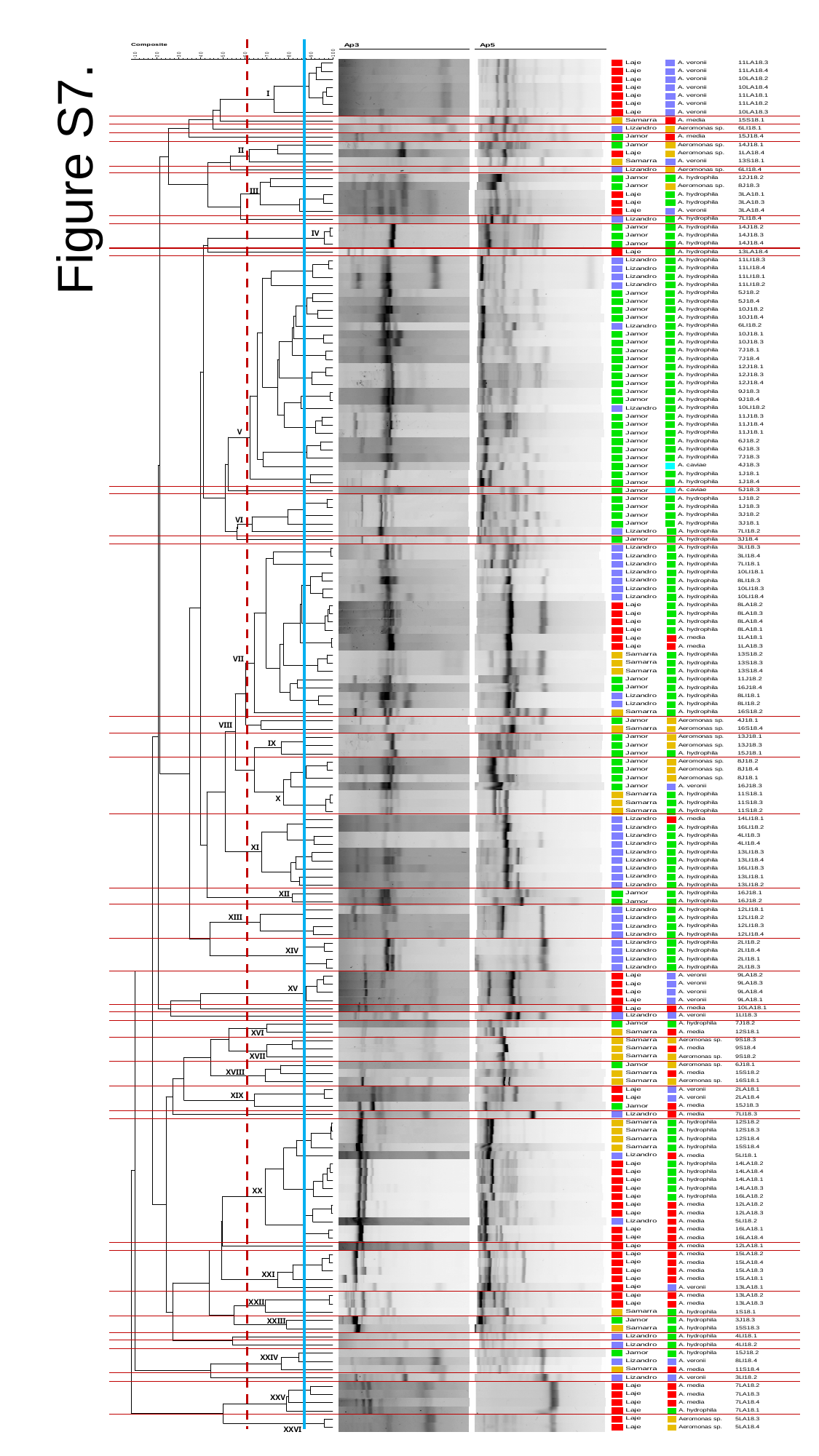

# Figure S7.
I
II
III
IV
V
VI
VII
VIII
IX
X
XI
XII
XIII
XIV
XV
XVI
XVII
XVIII
XIX
XX
XXI
XXII
XXIII
XXIV
XXV
XXVI
