# Supplementary material for: Molecular Epidemiology, Virulence Traits and Antimicrobial Resistance Signatures of Aeromonas spp. in the Critically Endangered Iberochondrostoma lusitanicum Follow Geographical and Seasonal Patterns
Source: Antibiotics (Basel). 2021 Jun 22;10(7):759. doi: 10.3390/antibiotics10070759 (PMC8300795; doi:10.3390/antibiotics10070759)
Supplement: Supplementary file 1 [file antibiotics-10-00759-s001.zip › Supplementary material 4.pptx]

## Slide 1
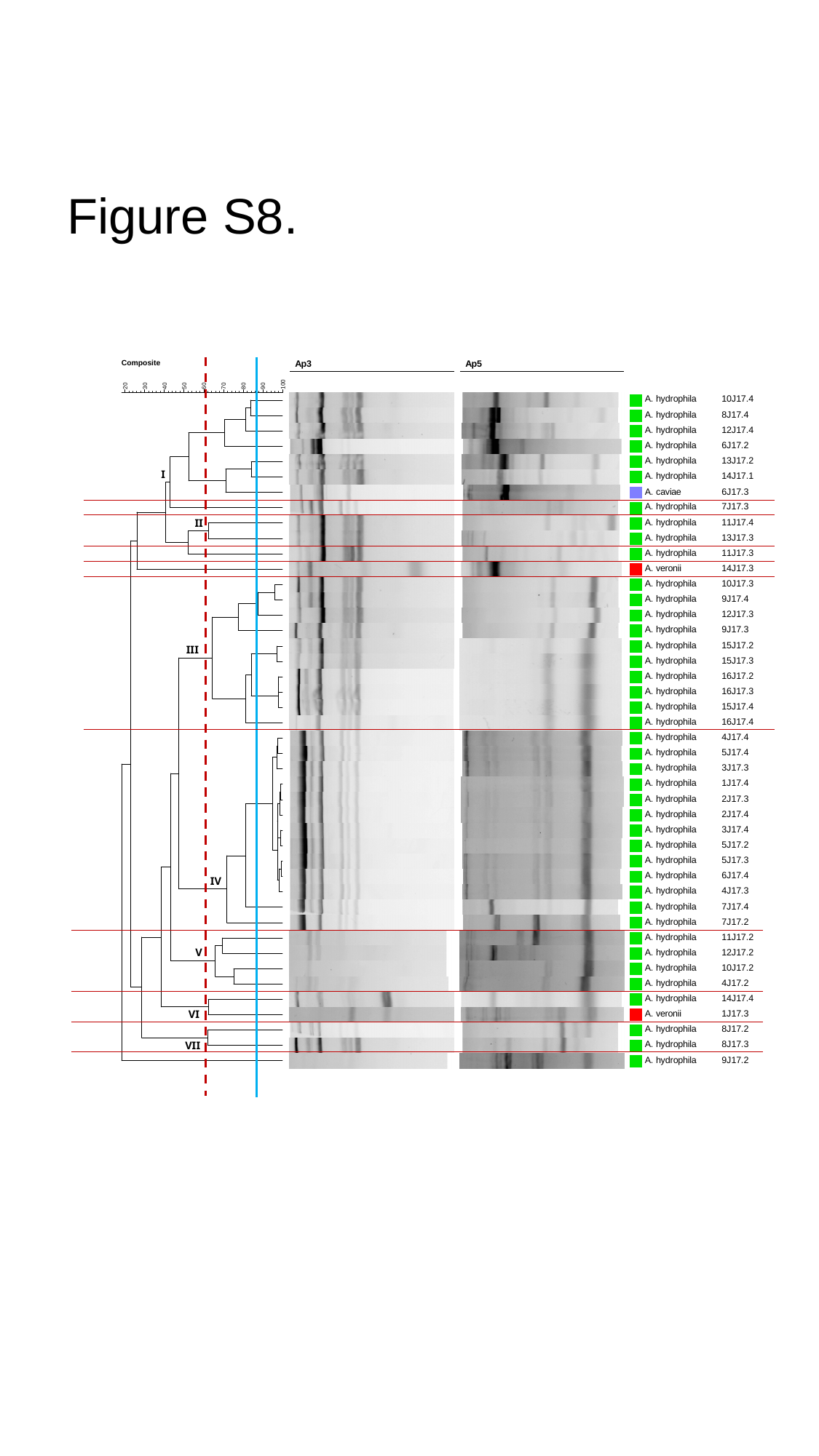

# Figure S8.
I
II
III
IV
V
VI
VII

## Slide 2
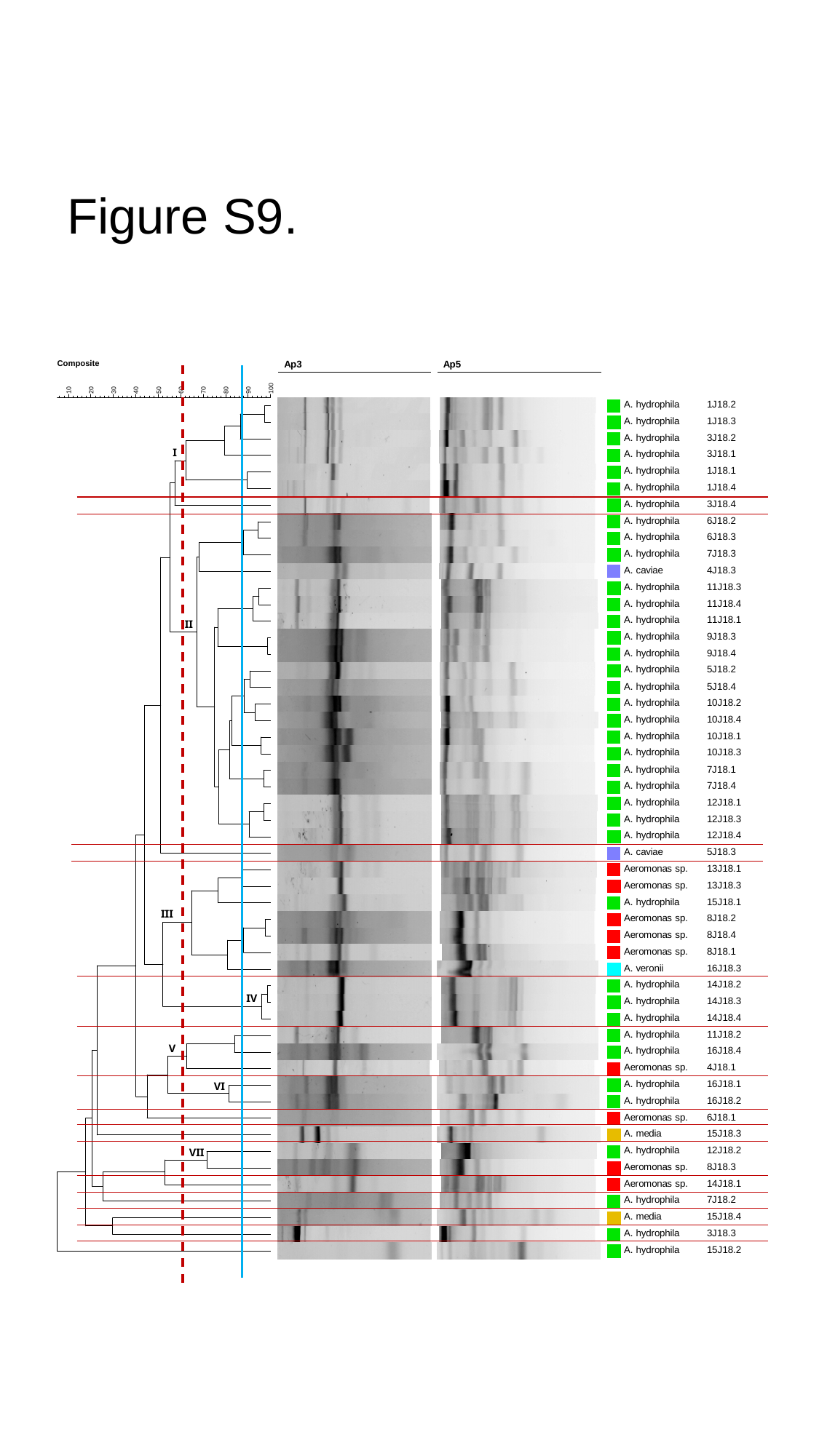

# Figure S9.
I
II
III
IV
V
VI
VII

## Slide 3
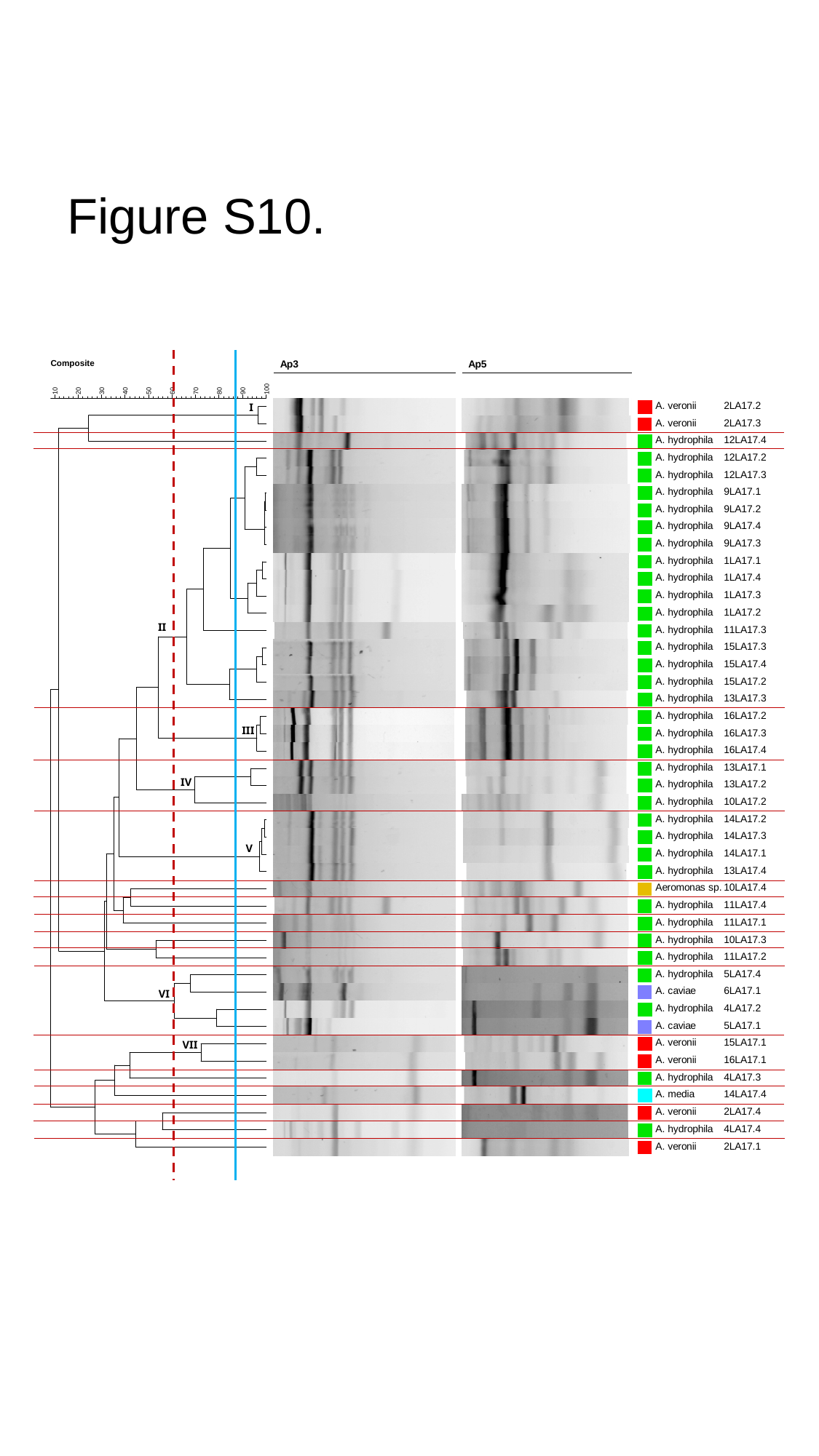

# Figure S10.
I
II
III
IV
V
VI
VII

## Slide 4
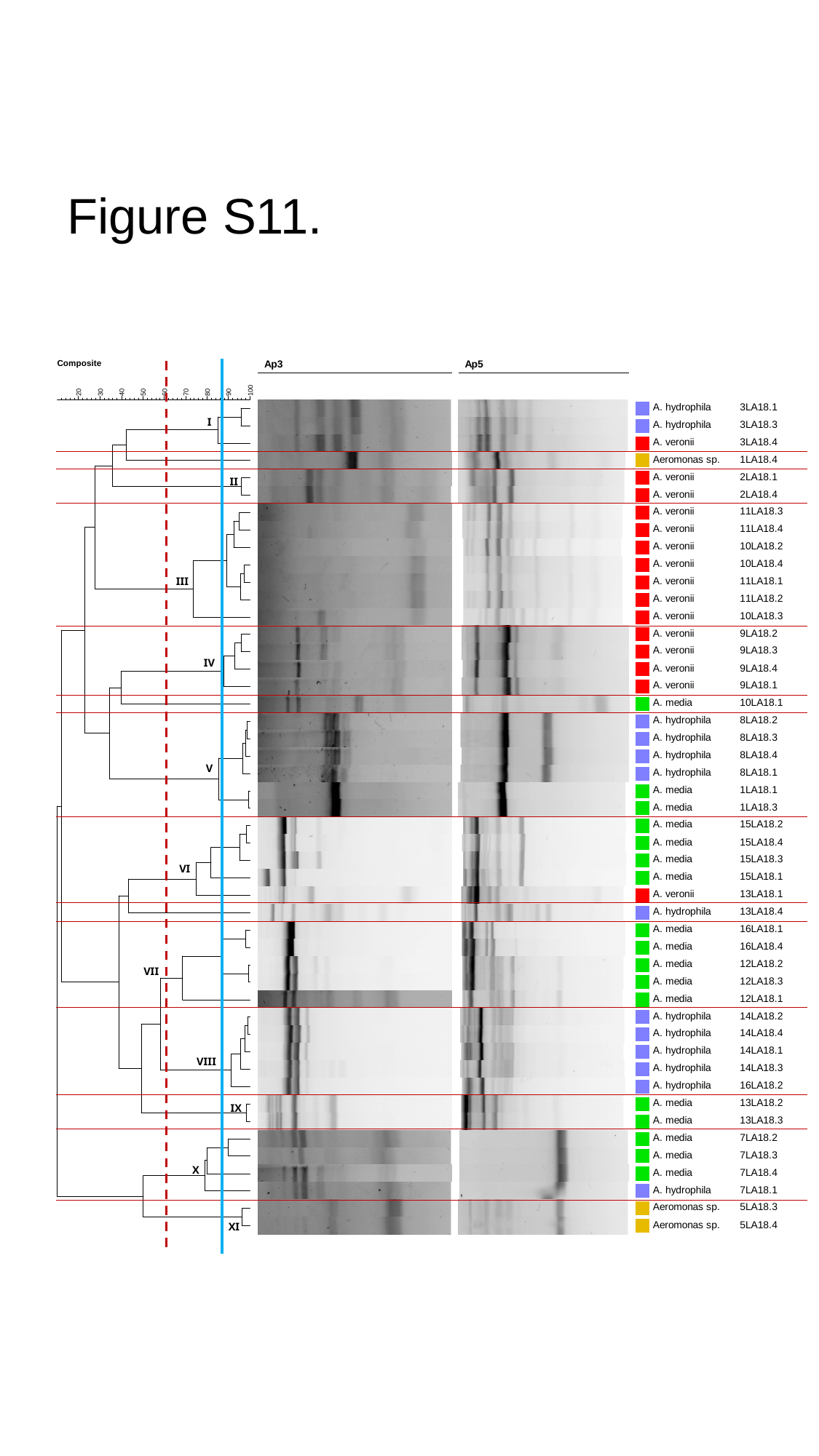

# Figure S11.
I
II
III
IV
V
VI
VII
VIII
IX
X
XI

## Slide 5
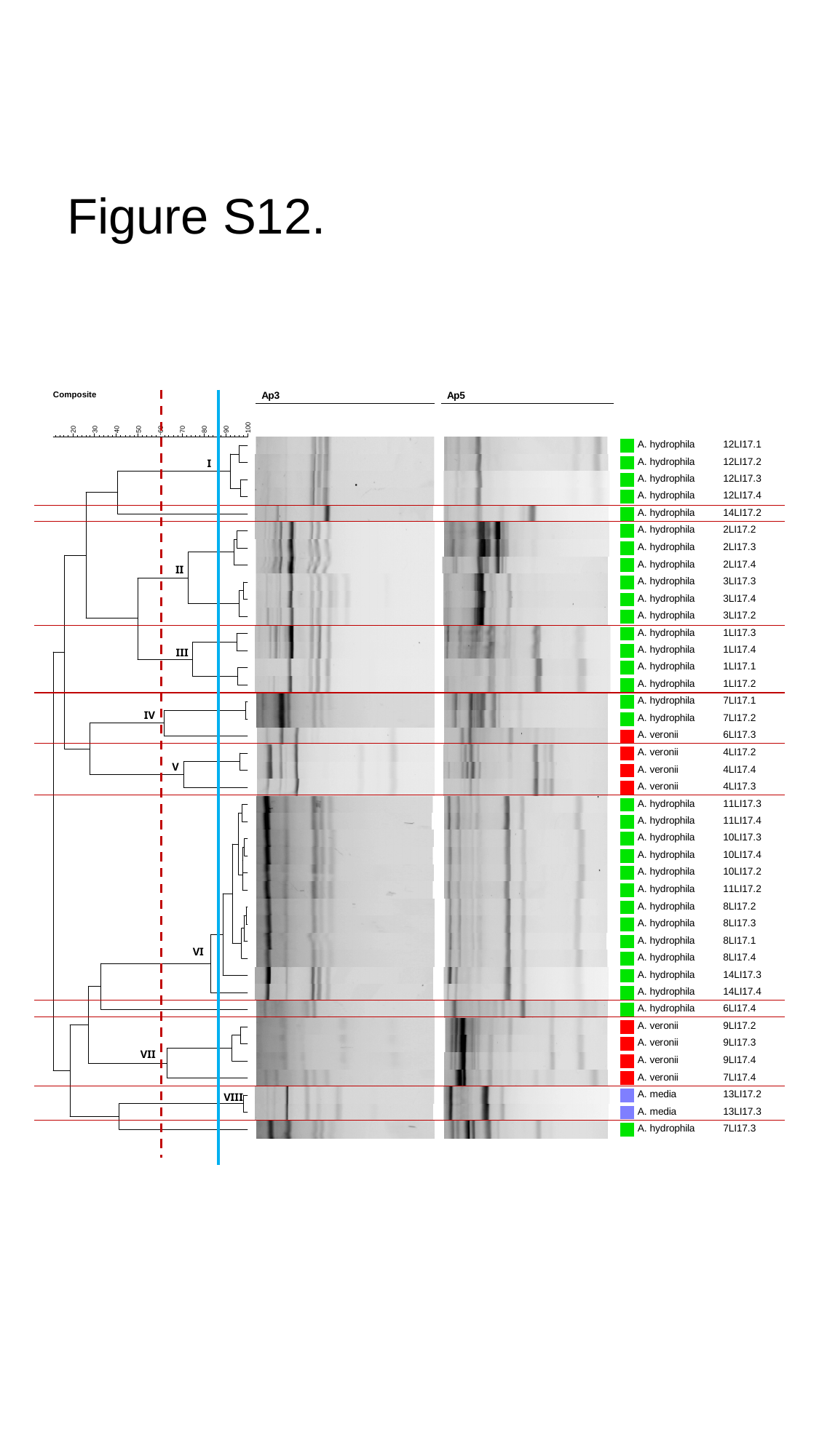

# Figure S12.
I
II
III
IV
V
VI
VII
VIII

## Slide 6
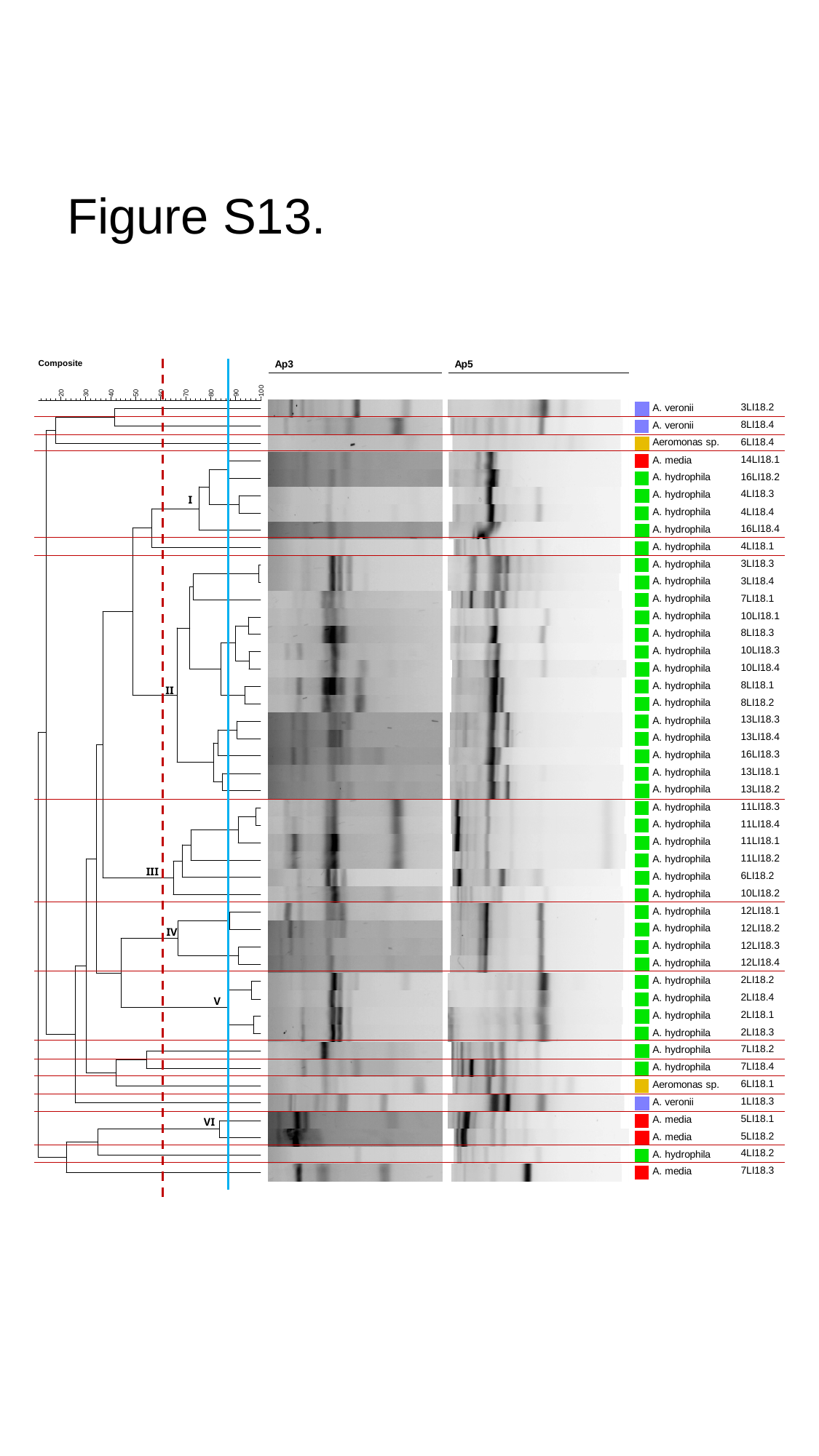

# Figure S13.
I
II
III
IV
V
VI

## Slide 7
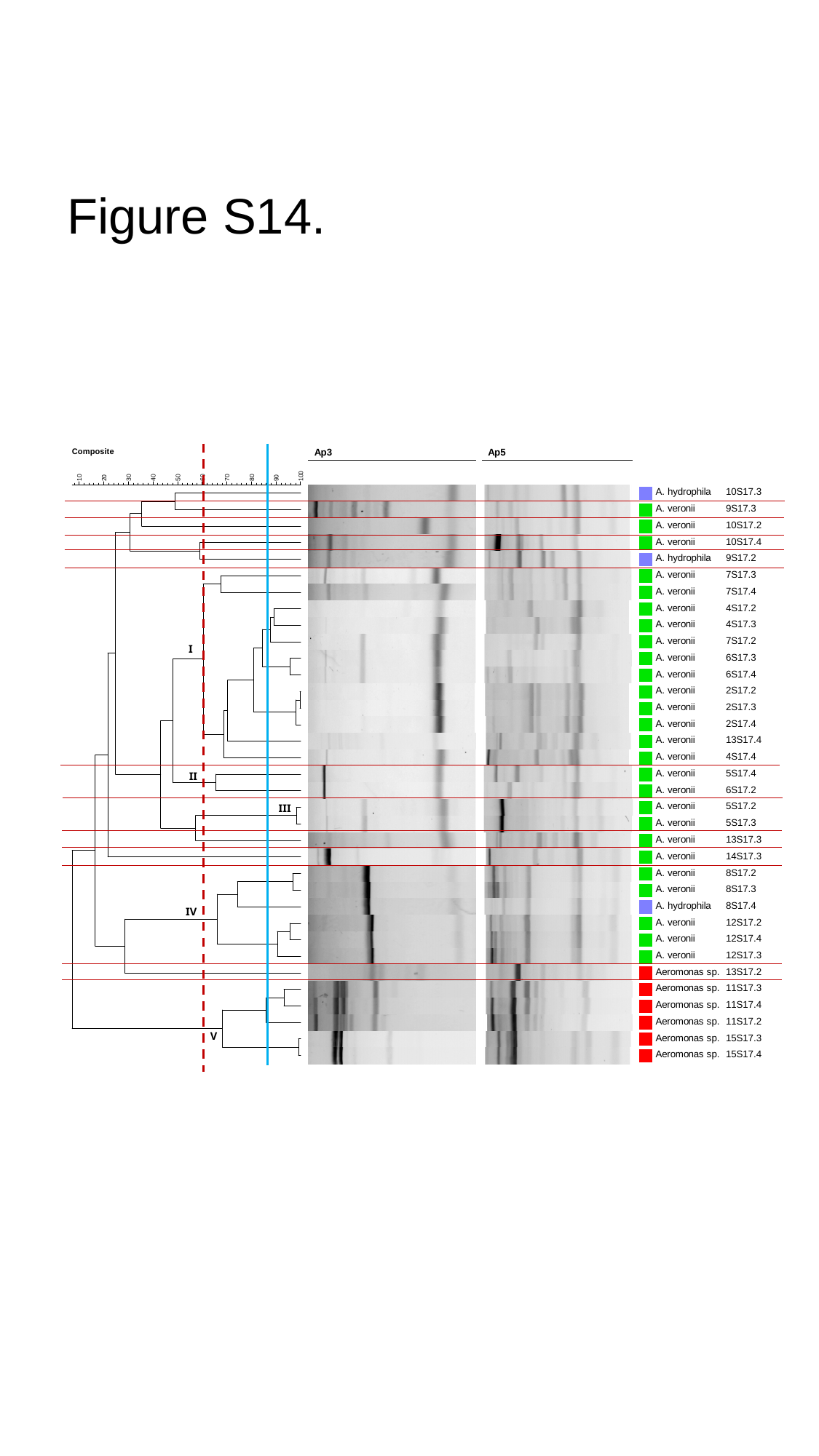

# Figure S14.
I
II
III
IV
V

## Slide 8
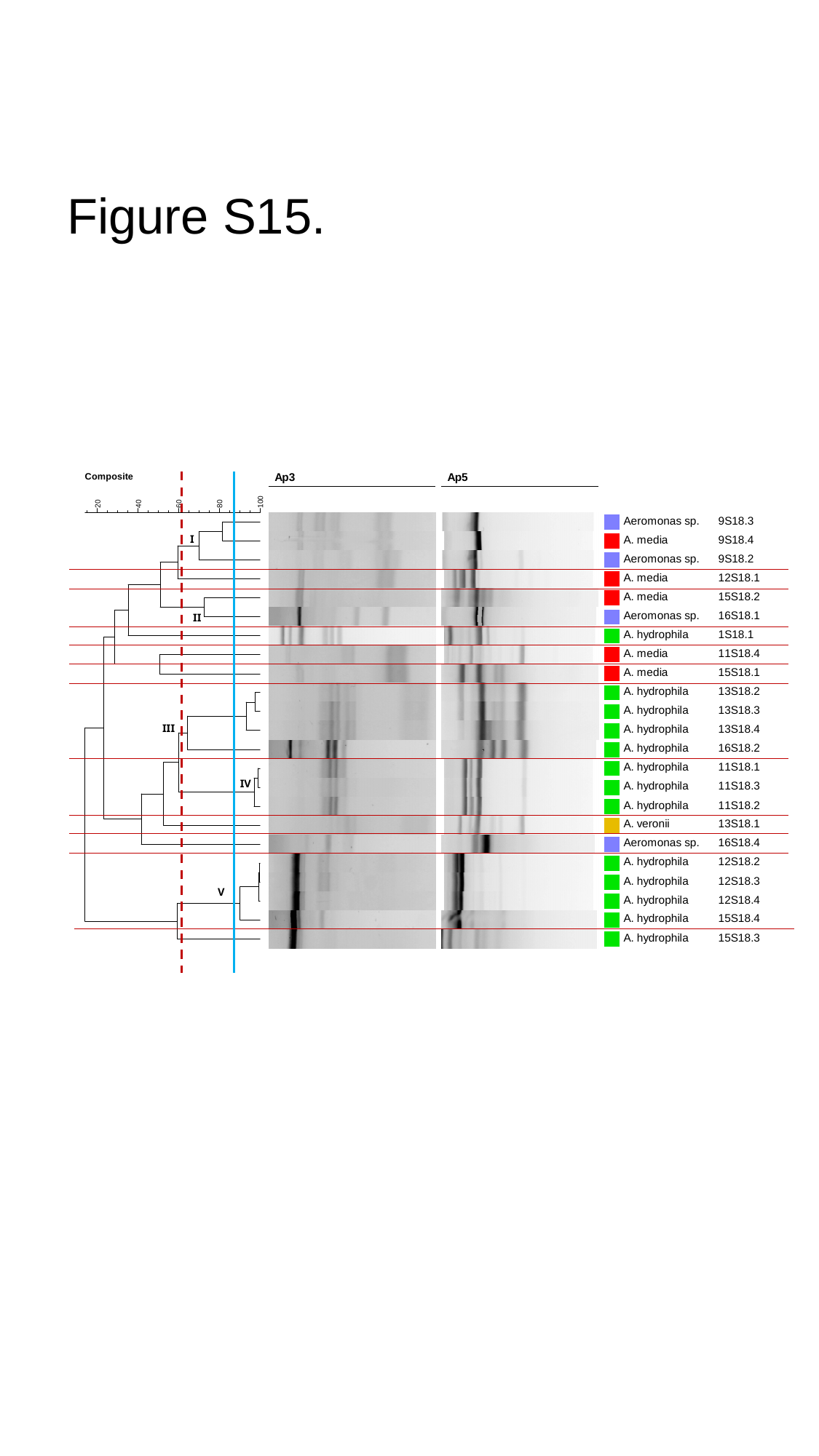

# Figure S15.
I
II
III
IV
V
